# Fumed and Precipitated Hydrophilic Silica Suspension Gels in Mineral Oil: Stability and Rheological Properties

**DOI:** 10.3390/gels3030032

**Published:** 2017-08-09

**Authors:** Yoshiki Sugino, Masami Kawaguchi

**Affiliations:** Laboratory of Colloid Rheology, Division of Chemistry for Materials, Graduate School of Engineering, Mie University, 1577 Kurimamachiya, Tsu 514-8507, Japan; y-sugino@co-jsp.co.jp

**Keywords:** fumed silica suspension, precipitated silica suspension, mineral oil, surface silanol density, stability, rheological properties

## Abstract

Hydrophilic fumed silica (FS) and precipitated silica (PS) powders were suspended in mineral oil; increasing the silica volume fraction (*φ* in the suspension led to the formation of sol, pre-gel, and gel states. Gelation took place at lower *φ* values in the FS than the PS suspension because of the lower silanol density on the FS surface. The shear stresses and dynamic moduli of the FS and PS suspensions were measured as a function of *φ*. Plots of the apparent shear viscosity against shear rate depended on *φ* and the silica powder. The FS suspensions in the gel state exhibited shear thinning, followed by a weak shear thickening or by constant viscosity with an increasing shear rate. In contrast, the PS suspensions in the gel state showed shear thinning, irrespective of *φ*. The dynamic moduli of the pre-gel and gel states were dependent on the surface silanol density: at a fixed *φ*, the storage modulus *G′* in the linear viscoelasticity region was larger for the FS than for the PS suspension. Beyond the linear region, the *G′* of the PS suspensions showed strain hardening and the loss modulus *G″* of the FS and PS suspensions exhibited weak strain overshoot.

## 1. Introduction

Pristine fumed silica (FS) powders produced by hydrolysis of silicone tetrachloride in a flame, such as hydrophilic or native fumed silica powders, have a low tendency to aggregate and to form agglomerates because of their lower surface density of silanol groups; this results in a lower bulk density compared with precipitated hydrophilic silica (PS) powders, produced by acid precipitation of sodium silicate. The properties of FS powders suspended in polymer liquids, such as polydimethylsiloxane (PDMS) [1,2,3,4,5,6,7,8,9,10] have often been compared with those of PS powders [11,12], and the following differences were identified. The preparation of FS suspensions in PDMS requires lower amounts of powders compared with PS suspensions (1); FS suspensions show a higher bound rubber content, defined as the amount of non-extractable PDMS rubber (expressed as a percentage of the initial PDMS amount), than PS suspensions; this suggests that the silica/PDMS interaction is stronger in FS than PS suspensions, with larger adsorbed amounts of PDMS in the former (2); in the case of FS suspensions, the maximum loss modulus was observed at the end of the linear viscoelasticity region, whereas the loss modulus (*G″*) vs. strain curves of the PS suspensions showed no indications of strain-induced agglomeration (3).

On the other hand, fewer studies have investigated PS suspensions in non-polar fluids such as mineral and paraffin oil, compared with several studies focused on FS suspensions. In the corresponding liquids, the FS particles interact through hydrogen bonds, which can result in the formation of a gel structure [13,14,15,16,17]. Khan and coworkers have thoroughly investigated the influence of the silica concentration on viscoelastic rheological properties [13,14,15] and static light scattering measurements [13] of FS suspensions in mineral oil, and they observed the presence of a gel-like network. Yziquel et al. have studied the non-linear viscoelastic behavior of FS suspensions in paraffin oil as a function of particle size and silica concentration [16]. They found that the reduced storage modulus was independent of the silica concentration and that the reduced loss modulus increased more rapidly with the strain amplitude when the silica concentration was increased. Chen et al. reported the rheological properties of FS suspensions in mineral oil as a function of particle size and silica concentration under steady and oscillatory shear [17]. They found that the relative viscosities of the FS suspensions were well fitted by the Krieger and Dougherty model [18], which is often applied to describe the relative viscosities of concentrated hard sphere suspensions and plays an important role in understanding their characteristics.

Although only few studies of PS suspensions in simple liquids, aimed at understanding their stability and rheological properties, have been reported, it can be expected that PS suspensions in non-polar fluids should be stable at higher silica concentrations because of their higher surface silanol density compared with the FS powders. Herein, we investigate changes in the phase state and rheological properties of FS and PS suspensions in mineral oil where the PS suspensions should form more compact aggregated microstructures than the FS suspensions due to the stronger hydrogen bonding between the higher surface silanol groups, associated with variations in the silica concentration. The results are discussed in terms of the different surface silanol density of the two materials.

## 2. Results and Discussion

### 2.1. Sample Classification

**Figure 1:**
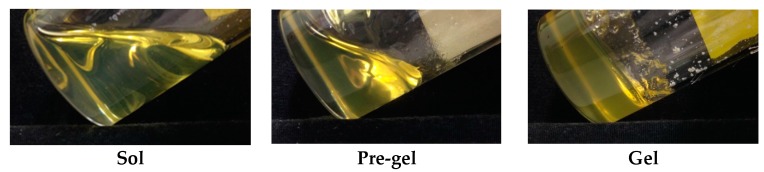
Visual appearance of the fumed silica (FS) suspensions in the sol, pre-gel, and gel states.

Based on visual inspection of the samples, the FS and PS suspensions were classified into sol, pre-gel, and gel states, irrespective of the silica powder type. The appearance of the silica suspensions depended on the surface silanol density: the FS suspensions were found in the sol state (for *φ* ≤ 0.0083, where *φ* is the volume fraction of silica in the suspensions), in the pre-gel state (for 0.0083 < *φ* ≤ 0.013), and in the gel state (for *φ* > 0.013); whereas the PS suspensions were in the sol state for *φ* ≤ 0.026, in the pre-gel state for 0.026 < *φ* ≤ 0.030, and in the gel state for *φ* > 0.030. Figure 1 shows typical visual appearances in the sol, pre-gel, and gel states of the FS suspensions. Gelation of the FS suspensions took place at lower silica concentrations compared with PS suspensions in mineral oil due to the less surface silanol density and the less compact aggregated microstructures. A similar concentration dependence of the gelation of PS suspensions in benzyl alcohol was observed by Hayashi and Kawaguchi [19].

### 2.2. Transient Shear Stress

**Figure 2:**
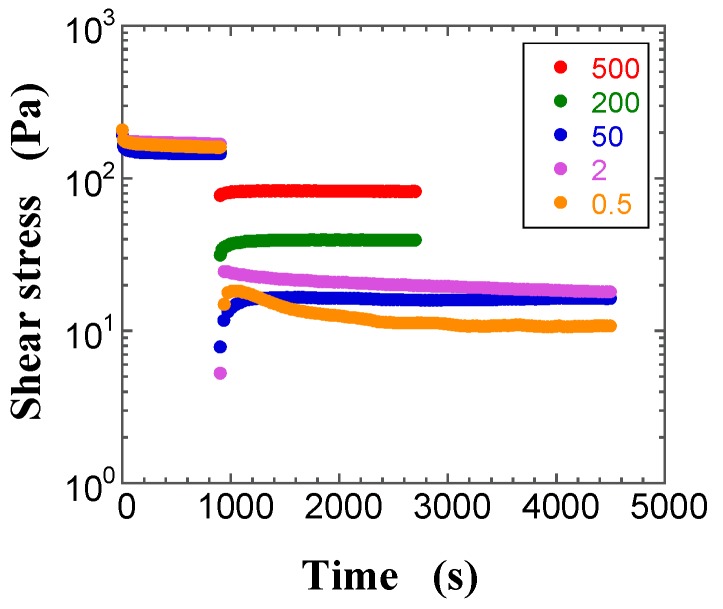
Plots of the transient shear stress for the FS suspension at the volume fraction of silica *φ* = 0.013 at the shear rates of 0.5 s^−1^ (filled orange circle), 2 s^−1^ (filled purple circle), 50 s^−1^ (filled blue circle), 200 s^−1^ (filled green circle), and 500 s^−1^ (filled red circle) as a function of time.

**Figure 3:**
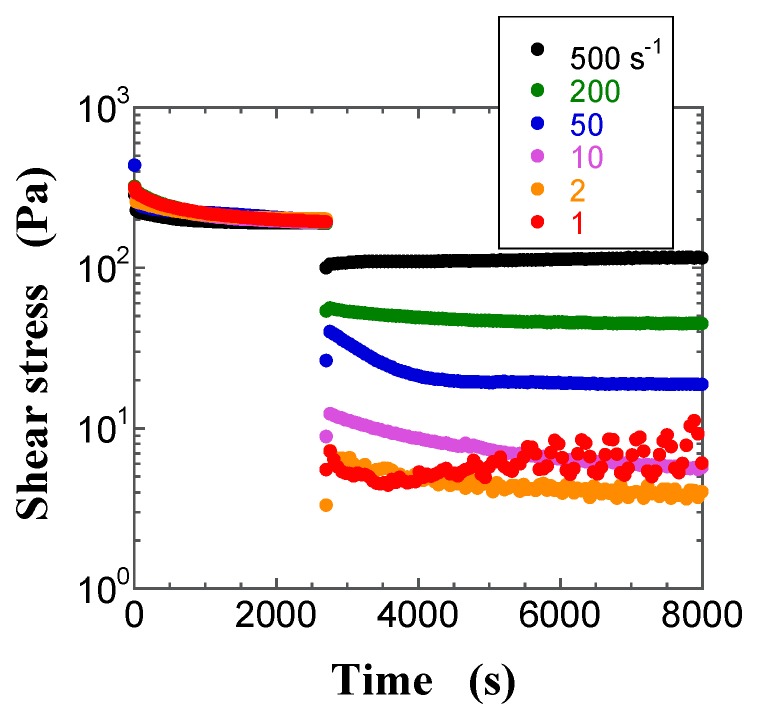
Plots of the transient shear stress for the FS suspension at *φ* = 0.017 at the shear rates of 1 s^−1^ (filled red circle), 2 s^−1^ (filled orange circle), 10 s^−1^ (filled purple circle), 50 s^−1^ (filled blue circle), 200 s^−1^ (filled green circle), and 500 s^−1^ (filled black circle) as a function of time.

**Figure 4:**
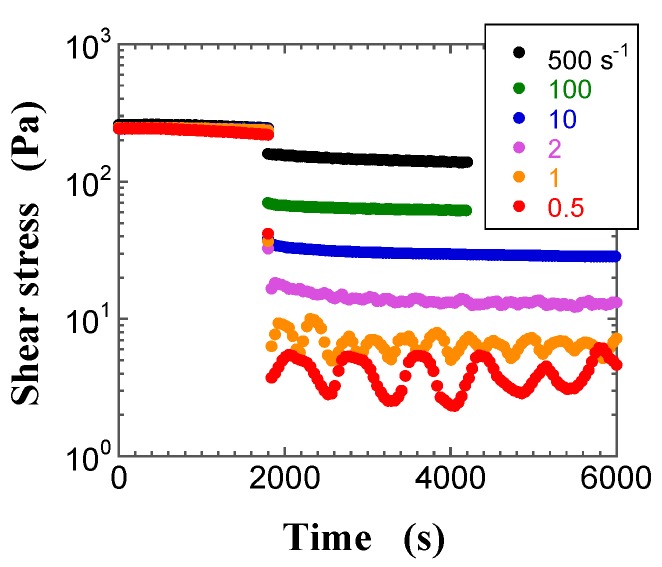
Plots of the transient shear stress for the precipitated silica (PS) suspension at *φ* = 0.030 at the shear rates of 0.5 s^−1^ (filled red circle), 1 s^−1^ (filled orange circle), 2 s^−1^ (filled purple circle), 10 s^−1^ (filled blue circle), 100 s^−1^ (filled green circle), and 500 s^−1^ (filled black circle) as a function of time.

Since a suspension is a complex dispersed system, it is important to understand its transient behavior before examining its steady-state response for measuring rheological properties. Figure 2, Figure 3 and Figure 4 show typical transient shear stress curves of the FS suspensions with *φ* = 0.013 (pre-gel state) and 0.017 (gel state), and of the PS suspension with *φ* = 0.030 (pre-gel state), respectively. During the preshearing period for the pre-gel state, the shear stress of the FS suspensions rapidly reaches the plateau, whereas the shear stress of the PS suspensions decreases with increasing time and then gradually levels off to a plateau value. After the preshearing period, Figure 2 and Figure 3 highlight a sigmoid increase of the shear stress with increasing time, whereas the shear stress decreases sigmoidally with an increase in time in Figure 4, irrespective of the shear rate ($\dot{\gamma }$). Similar changes in the measured shear stress after preshearing were obtained for the FS and PS suspensions, irrespective of *φ*. At shear rates lower than 2 s^−1^, the shear stress in Figure 3 and Figure 4 shows sustained oscillations, whose amplitude decreases with increasing shear rate. Moreover, sustained oscillations were observed for the FS suspensions in the gel state and for the PS suspensions in the pre-gel and gel states. In contrast, no sustained shear stress oscillations were observed for the FS suspensions in the pre-gel state, as shown in Figure 2. The difference in the FS and PS suspensions that exhibit sustained oscillations is likely related to their different surface silanol density. Sustained oscillations in shear stress may reflect continuous repeated changes in buildup and breakdown of microstructures in the aggregated silica suspensions.

Similar sustained oscillations in the shear stress were observed for silica suspensions [20,21], wormlike micelle solutions [22,23], as well as lamellar, onion, and sponge phases of surfactants [24,25]. In these cases, the sustained oscillations were observed at specific shear rate ranges, beyond which steady-state shear thinning or thickening was observed.

### 2.3. Steady-State Shear Viscosity

**Figure 5:**
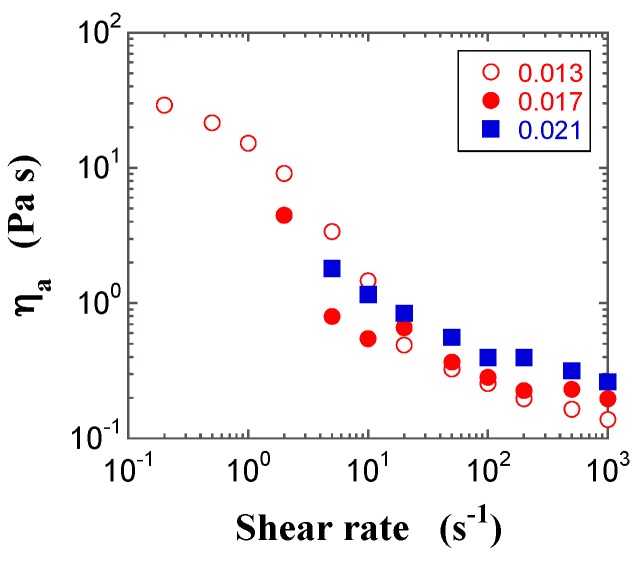
Double-logarithmic plots of the apparent steady-state shear viscosities (*η*_a_) for the FS suspensions at *φ* = 0.013 (open red circle), 0.017 (filled red circle), and 0.021 (filled blue square) as a function of the shear rate.

**Figure 6:**
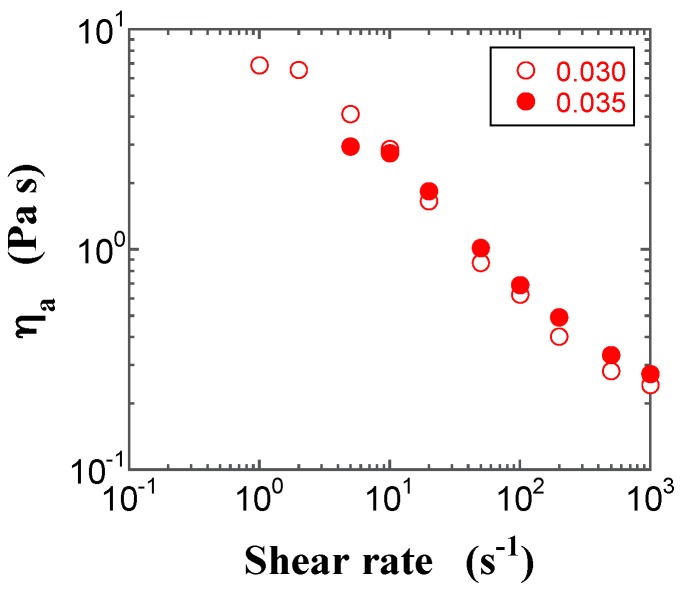
Double-logarithmic plots of the apparent steady-state shear viscosities for the PS suspensions at *φ* = 0.030 (open red circle) and 0.035 (filled red circle) as a function of the shear rate.

Typical plots of the apparent steady-state shear viscosity (*η*_a_) calculated from the ratio of the steady-state shear stress and $\dot{\gamma }$, as a function of shear rate for FS and PS suspensions with different *φ* values, are shown in Figure 5 and Figure 6, respectively. The steady-state shear stress was regarded when the transient shear stress was almost independent of time for at least 5 min during the transient sweep. The plot for the FS suspension in the pre-gel state displays shear thinning behavior, while the FS suspension with *φ* = 0.017 in the gel state exhibits shear thinning behavior, followed by a small bump in *η*_a_ at the shear rate of 20 s^−1^, which seems to be a kind of weak shear thickening, and the FS suspension with *φ* = 0.030 in the gel state shows shear thinning behavior, followed by constant and decreasing *η*_a_ with an increasing shear rate. In contrast, the PS suspensions only exhibit shear thinning behavior, irrespective of the specific state. The difference in *η*_a_ values between the FS and PS suspensions depends on the surface silanol density. On the other hand, when FS powders were dispersed in polar organic solvents, some interesting shear thickening behaviors were observed and their characteristics were discussed by the interactions between silica particles and dispersants [14,15,26,27,28].

A useful parameter to investigate the aggregated structure of particles suspended in a dispersing liquid is the relative viscosity (*η*_r_) defined as the ratio of *η*_a_ to the viscosity of the dispersing liquid (*η*_o_). Chen et al. reported that the *η*_r_ data of hydrophilic and hydrophobic FS suspensions in mineral oil [17] were well fitted by the Krieger and Dougherty model [18], which is often applied to describe the relative viscosities of concentrated hard sphere suspensions and plays an important role in understanding their characteristics. Hayashi and Kawaguchi [19] also reported that the *η*_r_ values of hydrophobic PS suspensions in benzyl alcohol were in good agreement with the Krieger and Dougherty model [18].

**Figure 7:**
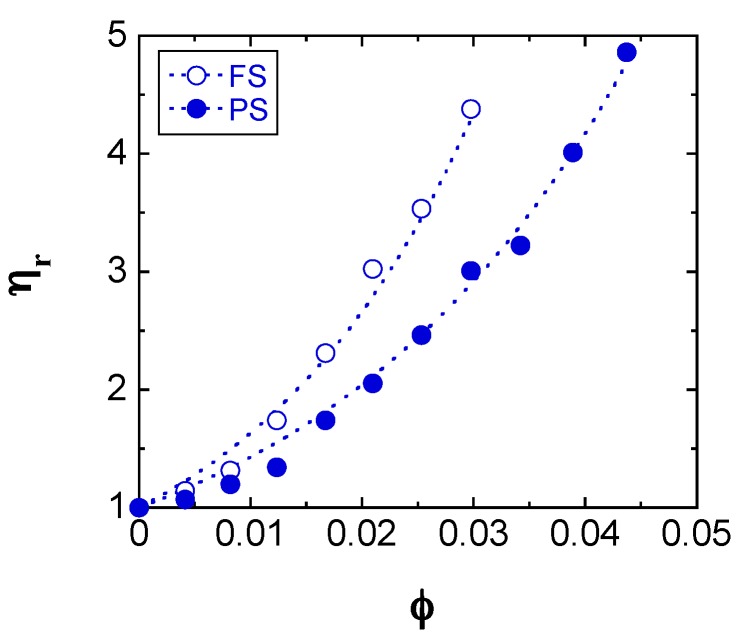
The relative viscosity values of the FS (open blue circle) and PS suspensions (filled blue circle) at the shear rate of 10^3^ s^−1^ as a function of *φ*. The dashed lines represent fit to the Krieger and Dougherty equation.

The Krieger and Dougherty model is described by the following equation:

(1) $$\eta_{r}={\left(1-\frac{\varphi }{\varphi_{m}}\right)}^{-\left[\eta \right]\varphi_{m}}$$

where *φ*_m_ is the maximum packing fraction and [η] is the intrinsic viscosity of the suspension. Figure 7 shows the fitting of the Krieger and Dougherty equation to the *η*_r_ vs. *φ* values measured for the FS and PS suspensions at a shear rate of 1000 s^−1^; the fitting was performed by the least squares method with two unknown parameters: *φ*_m_ and [η]. The dashed lines in the figure, corresponding to the Krieger and Dougherty equation, show an excellent fit to the experimental data, with resulting *φ*_m_ and [η] parameters of 0.41 and 48 for the FS suspensions and 0.51 and 34 for the PS suspensions, respectively. The fitted *φ*_m_ values are smaller than the theoretical value of 0.74 for a close-packed array of spheres of identical size, and the *φ*_m_ obtained for the FS suspensions is lower than that corresponding to the PS suspensions. This indicates that the FS and PS suspensions have a relatively more open structure than the concentrated hard sphere suspensions, and the PS suspensions form more tightly packed and agglomerated structures than the FS ones, owing to stronger hydrogen bonding interactions [11,12,19]. In contrast, the [η] value of the FS suspensions is larger than that of the PS ones, indicating that the former suspensions are less compact and form larger agglomerated structures than the latter. Thus, larger [η] values correspond to less compacted aggregates [29].

### 2.4. Dynamic Modulus

**Figure 8:**
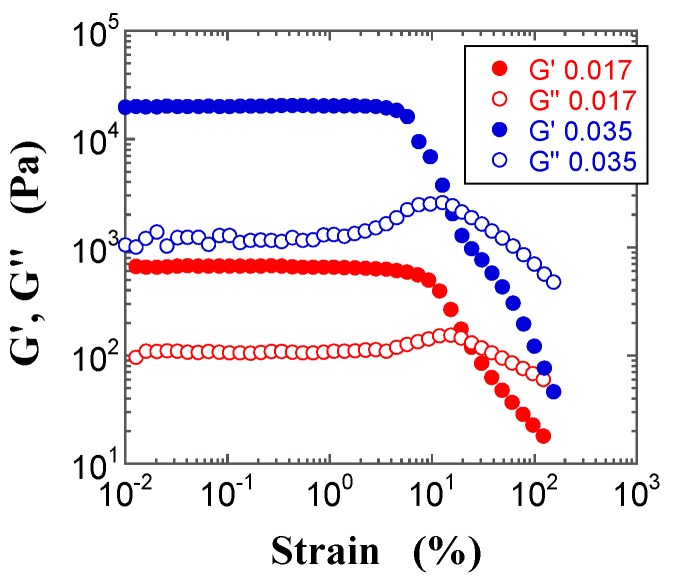
Double-logarithmic plots of the storage modulus (*G′*, filled circles) and the loss modulus (*G″*, open squares) for the FS suspensions at *φ* = 0.017 (red) and 0.035 (blue) as a function of strain.

**Figure 9:**
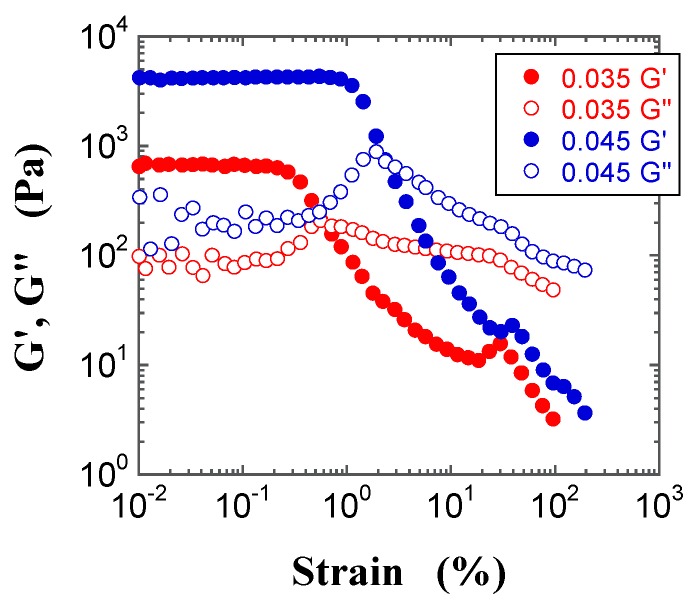
Double-logarithmic plots of *G′* (filled circles) and *G″* (open circles) for the PS suspensions at *φ* = 0.035 (red) and 0.045 (blue) as a function of strain.

Typical dynamic modulus vs. oscillatory shear strain curves of FS and PS suspensions with various *φ* values are shown in Figure 8 and Figure 9, respectively. The figures highlight that the linear region, in which the dynamic modulus is independent of the oscillatory strain, spans a wider range for the FS than the PS suspension; moreover, the linear region of the FS suspension decreases with increasing *φ*, whereas that of the PS suspension shows the opposite *φ* dependence. In addition, at *φ* = 0.035, the dynamic modulus of the FS suspension in the linear region is larger than that of the PS suspension. This suggests that the structure of the FS suspension is stronger and harder to break up than that of the PS suspension. The observed differences in the dynamic moduli and in the corresponding range of their linear regions likely derive from the somewhat stronger attractive interactions between aggregated silica particles in mineral oil with decreasing surface silanol density, as further discussed below. Similar results were observed for all *φ* values, and the dynamic moduli in the linear region increased with increasing *φ*, irrespective of the silica powder. Moreover, the storage modulus (*G′*) in the linear region was larger than the loss modulus (*G″*), irrespective of *φ*.

The presence of the linear region in the oscillatory shear tests indicates that the FS and PS suspensions in the gel state behave as a gel network, formed upon coalescence of aggregates of silica particles (i.e., a floc). The latter process is promoted by bonding interactions between the silica particles, which take place through the hydrogen bonds in the mineral oil. The formation of the gel network in the silica suspensions can be analyzed through the silica concentration dependence of the *G′* value in the linear region (*G′*_0_), and of the critical oscillatory shear strain (*γ*_c_) at which the linear region ends, according to scaling models based on fractal analysis [30,31,32]. This approach has been successfully applied to several systems, such as FS suspensions in organic solvents [16,33,34,35] and in water [36,37,38], and PS suspensions in benzyl alcohol [19].

**Figure 10:**
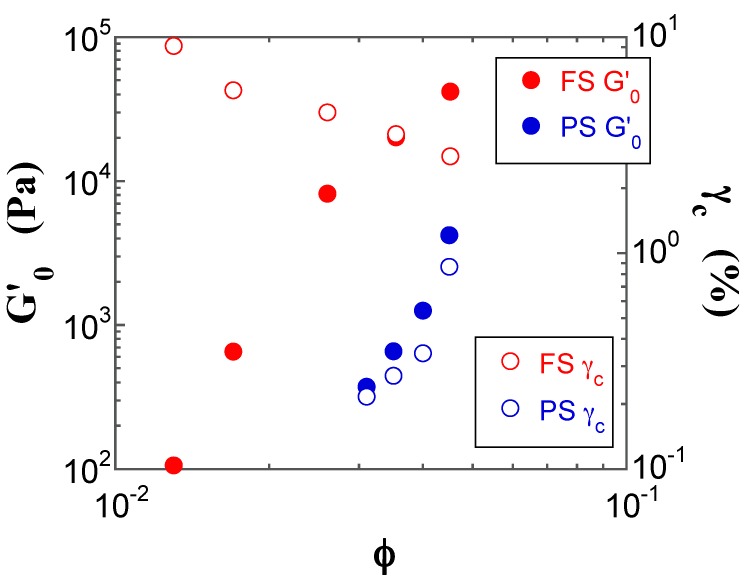
Double-logarithmic plots of the *G′* value in the linear region (*G′*_0_, filled circles) and the critical oscillatory shear strain (*γ*_c_, open circles) for the FS (red) and PS (blue) suspensions as a function of *φ*.

As shown in Figure 10, *G′*_0_ increases with increasing *φ* for both FS and PS suspensions; in contrast, the magnitude of the *γ*_c_ value of the FS and PS suspensions decreases and increases with increasing *φ*, respectively. Such *φ* dependences indicate that the FS and PS suspensions can be classified as a strong-link and a weak-link gel, respectively [30,31,32]. In the case of a strong-link gel, the links between different flocs (interfloc links) have a higher elastic constant than those within the same floc (intrafloc links), whereas in a weak-link gel the interfloc links are weaker than the intrafloc ones [30,31,32]. This shows that the PS suspensions form more compact flocs than the FS suspensions due to the higher surface silanol density. The difference in the gel state associated with the FS and PS silica suspensions leads to their unique linear rheological responses described above.

Although it is possible to estimate the fractal dimension of the FS and PS suspensions from the slope of the double logarithmic plots of *G′*_0_ and *γ*_c_ as a function of *φ*, we did not attempt to calculate it in this case, owing to the narrow ranges of *φ* in the pre-gel and gel states, as well as to the somewhat scattered data (see Figure 10).

Furthermore, from the frequency dependence of the dynamic moduli, the linear region—observed for the FS and PS suspensions in the pre-gel and gel states (not shown)—indicates that the *G′* and *G″* values are almost independent of the frequency, irrespective of *φ*, and the magnitudes of the *G′* values are larger than those of the *G″* values. This indicates that these silica suspensions are in the gel state.

The non-linear trends beyond the linear region in the dynamic moduli vs. strain plots, known as large amplitude oscillatory shear (LAOS) responses, provide information on the interactions and on the shear-induced formation of microstructures in complex fluids, such as polymer solutions, polymer melts, suspensions, and emulsions. Hyun et al. [39,40,41] proposed that the LAOS responses can be classified into four different categories: strain thinning, strain hardening, weak strain overshoot, and strong strain overshoot. Figure 8 and Figure 9 highlight some significant features in the LAOS responses of the FS and PS suspensions. In the case of the FS suspensions, the *G′* values beyond the linear region decrease with increasing strain, whereas the *G″* values show a weak strain overshoot; the *G″* and *G′* curves cross each other at a given strain, and then gradually decrease with increasing strain. The strain at which the *G″* and *G′* curves cross each other decreases with increasing *φ*, which indicates that the FS suspensions become more brittle with increasing *φ*. An increase in *G″* just before decreasing in the non-linear region seems to correspond to a dissipative energy due to partial deformation of aggregated microstructures under shear. Similar results were obtained for several silica suspensions [14,15,19,33,34,35].

In contrast, the *G′* values for the PS suspensions beyond the linear region decrease less steeply with increasing strain, compared with the FS suspensions. The *G′* values show a clear peak, namely strain hardening at a strain value that increases with increasing *φ*, and the *G″* values also exhibit a weak strain overshoot: in particular, the PS suspensions exhibit a strong strain overshoot. The *G″* and *G′* curves cross each other at a given strain, and then gradually decrease with increasing strain. The strain values corresponding to the strain hardening in the *G′* values and to the crossover point between the *G″* and *G′* curves increase with increasing *φ* this indicates that the PS suspensions possess stronger structures than the FS suspensions.

The different *φ* dependence of the strain value at which the crossover occurs in the FS and PS suspensions and the presence of strain hardening in the *G′* values of the PS suspensions seem to indicate that the PS suspensions form more rigid and less brittle aggregated microstructures than the FS ones under the conditions corresponding to the LAOS responses. Moreover, the strains corresponding to the crossover point are higher for the present FS suspensions than for FS suspensions in benzyl alcohol [33], for which the *G′*_0_ values range from 4 × 10^3^ to 2 × 10^4^ Pa, and the corresponding strains are less than 1% in the gel states with 0.095 ≤ *φ* ≤ 0.115.

**Figure 11:**
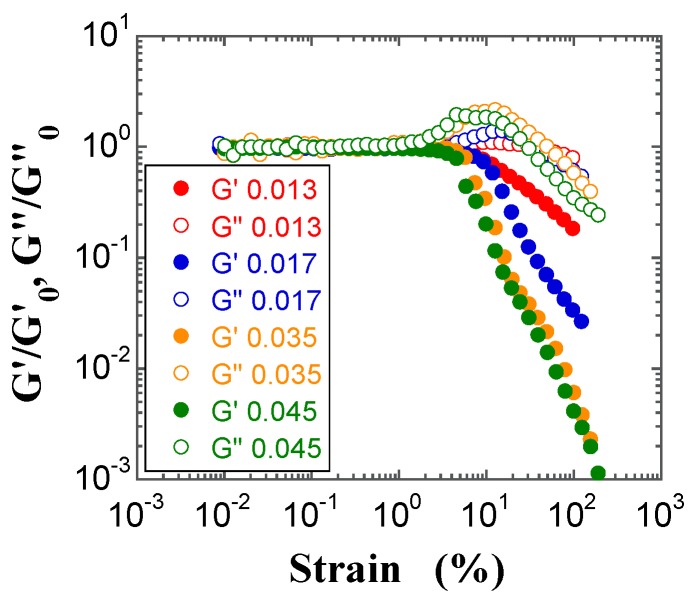
Double-logarithmic plots of *G′/G′*_0_ (filled circles) and *G″*/*G″*_0_ (open circles) for the FS suspensions at *φ* = 0.013 (red), 0.017 (red), 0.035 (orange), and 0.045 (green) as a function of strain. *G″*_0_ is the loss modulus in the linear region.

**Figure 12:**
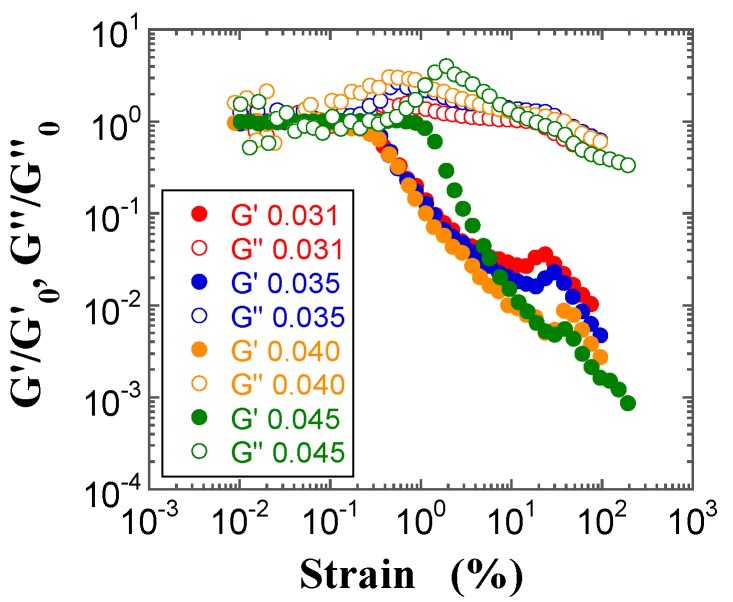
Double-logarithmic plots of *G′/G′*_0_ (filled circles) and *G″*/*G″*_0_ (open circles) for the PS suspensions at *φ* = 0.031 (red), 0.035 (red), 0.040 (orange), and 0.045 (green) as a function of strain.

Furthermore, the double logarithmic plots of the reduced moduli (*G′*/*G′*_0_ and *G″*/*G″*_0_, where *G″*_0_ is the loss modulus in the linear region) vs. strain in the LAOS response region have often proven useful to understand the complicated behavior of complex fluids [39,40,41], as well as of FS [34,35] and PS silica suspensions [19]. The reduced moduli *G′*/*G′*_0_ and *G″*/*G″*_0_ for the FS and PS suspensions are plotted in Figure 11 and Figure 12, respectively. Both reduced moduli do not fully overlap with the respective master curves: the *G′*/*G′*_0_ values show a negative deviation that increases with increasing *φ*; this occurs at strain values higher than 5% for the FS suspensions and beyond the stain hardening for the PS suspensions, respectively. On the other hand, the *G″*/*G″*_0_ values beyond the strain overshoot show a negative deviation that increases with increasing *φ*. Similar negative deviations of the reduced *G′*/*G′*_0_ and *G″*/*G″*_0_ moduli were observed for FS suspension gels in benzyl alcohol [33]. In contrast, a positive deviation of the *G″*/*G″*_0_ values beyond the strain overshoot has often been observed for hydrophobic FS and PS suspensions in organic solvents [19,34,35], where strong hydrophobic interactions between hydrophobic chains and the dispersion medium are present.

## 3. Experimental Procedures

### 3.1. Materials

FS and PS powders were kindly supplied from Nippon Aerosil Co. Ltd. (Yokkaichi, Japan) and Tosoh Silica Co. Ltd. (Shunan, Japan), respectively. The primary silica particle size, Brunauer–Emmett–Teller (BET) specific surface area, and surface silanol density of the FS powders were 16 nm, 130 ± 25 m^2^/g, and 2.2 nm^−2^, respectively. On the other hand, the PS powders exhibited a primary silica particle size of 19 nm, a BET specific surface area of 155 m^2^/g, and a surface silanol density of ca. 4 nm^−2^. The above product specifications were obtained from the respective suppliers. The respective silica powders were dried under vacuum in an incubator box and then stored until the preparation of their suspensions.

Mineral oil Cosmo Pure Safety 46, supplied from Cosmo Oil Lubricants Co. Ltd. (Tokyo, Japan), was used as a dispersant, without further purification. This mineral oil has a density of 0.895 g cm^−3^ at 15 °C, a kinematic viscosity of 43.5 cSt (cm^2^ s^−1^) at 40 °C, an average molecular weight of 403 g mol^−1^, and paraffinic, naphthenic, and aromatic hydrocarbon contents of 59.2, 30.2, and 10.6 wt %, respectively.

### 3.2. Preparation of Silica Suspensions

To prepare a suspension, weighted amounts of the silica powder and 30 mL mineral oil were mixed in a 150 mL vessel. The silica concentrations were expressed by *φ* of silica in the suspensions. The resulting suspensions were agitated for 5 min with a rotation of 800 rpm and a revolution of 2000 rpm, and then de-aerated for 3 min with a rotation of 60 rpm and a revolution of 2200 rpm using a Keyence (Thinky Corporation, Tokyo, Japan) HM-500 hybrid mixer—A planetary centrifugal mixer that uses a mechanism in which the container holding the material revolves clockwise and the container itself rotates counter-clockwise. The suspensions were placed in a Sanyo MIR-153 incubator (Sanyo Electric Co., Ltd., Osaka, Japan) at 25 °C for 1 h under double agitation, after which they were subjected to visual inspections and rheological measurements.

### 3.3. Rheological Measurements

The FS and PS suspensions in the pre-gel and gel states were placed on a frosted glass plate of a cone-plate fixture with a diameter of 35 mm and an angle of 1° and then preconditioned by shearing at a rate of 1000 s^−1^ for 1500 s, 2000 s, or 3600 s. Longer preconditioning times were needed with increasing *φ* The serrated plate was used to suppress slippage of the suspension at the sample/plate interface. Transient stress measurements using a stress-controlled Rheoscope 1 rheometer (Thermo Fisher Scientific, Waltham, MA, USA) at 25 °C were then performed. The time evolutions of the transient shear stress of the suspensions were measured at shear rates ranging from 0.1 to 1000 s^−1^. This procedure was usually carried out over a period of less than 60 min at 25 °C. When the transient shear stress did not reach a steady-state within the 30 min period, it took up to 90 min to measure the transient shear stress.

On the other hand, for the measurements of the dynamic viscoelastic moduli, the FS and PS suspensions in the pre-gel and gel states were placed in a parallel plate fixture with a diameter of 35 mm and a gap of 1 mm and in the same cone-plate fixture described above, respectively. The dynamic viscoelastic moduli measurements were carried out under oscillatory strains increasing from 0.1 to 1000% at 25 °C and a fixed frequency of 5 rad/s, using the same stress-controlled Rheoscope 1 rheometer. Moreover, the frequency sweep measurements were performed in the linear viscoelastic region at a frequency ranging from 0.1 to 100 rad/s and with a fixed shear strain of 0.1%. The rheological measurements were repeated at least three times, and the corresponding experimental errors were within 5%.

## 4. Conclusions

FS and PS powders were suspended in mineral oil, and the sol, pre-gel, and gel states of the resulting suspensions were investigated. The transient shear stress and steady-state shear viscosity of the silica suspensions in the pre-gel and gel states were investigated as a function of *φ*. At shear rates higher than 2 s^−1^, the silica suspensions almost approached a steady-state value within 30 min after the preshearing stage, irrespective of *φ*. At shear rates lower than 2 s^−1^, sustained oscillations in the shear stress were observed for the FS suspensions in the gel state and for the PS suspensions in the pre-gel and gel states. The *η*_r_ data of the FS and PS suspensions were well fitted by the Krieger and Dougherty model, and the fitted *φ*_m_ and [η] parameters indicate that the PS suspensions form more tightly packed and agglomerated structures than the FS suspensions. Based on dynamic modulus measurements in the linear region, the FS and PS suspensions could be classified as strong-link and weak-link gels, respectively, according to the fractal model. The LAOS responses highlighted a weak strain overshoot for the FS suspensions, whereas the PS suspensions showed strong strain overshoot. Therefore, an increase in the surface silanol density easily caused hydrogen bonding interactions between the surface silanol groups, formed more compact and smaller aggregated structures in the PS suspensions, and then the corresponding suspensions yielded lower shear stress and weaker dynamic moduli than the FS suspensions. Thus, the present study provides new data illustrating the influence of the surface silanol density on the shear stress and dynamic modulus data of silica suspension gels in mineral oil because the surface silanol density of the PS powders is twice as high as that of the FS powders.
